# Ruthenium oxide modified hierarchically porous boron-doped graphene aerogels as oxygen electrodes for lithium–oxygen batteries†

**DOI:** 10.1039/c8ra08763f

**Published:** 2018-11-29

**Authors:** Xiuhui Zhang, Xiang Chen, Chunguang Chen, Tie Liu, Mengmeng Liu, Congcong Zhang, Tao Huang, Aishui Yu

**Affiliations:** Department of Chemistry, Shanghai Key Laboratory of Molecular Catalysis and Innovative Materials, Institute of New Energy, Collaborative Innovation Center of Chemistry for Energy Materials, Fudan University Shanghai 200433 China; Laboratory of Advanced Materials, Shanghai Key Laboratory of Molecular Catalysis and Innovative Materials, Institute of New Energy, Collaborative Innovation Center of Chemistry for Energy Materials, Fudan University Shanghai 200433 China asyu@fudan.edu.cn

## Abstract

Suitable catalysts and reasonable structures for oxygen electrodes can effectively improve the electrochemical performance of lithium–oxygen batteries. In this work, ruthenium oxide modified boron-doped hierarchically porous reduced graphene aerogels (RuO_2_-B-HRG) are prepared by a sol–gel and subsequent low temperature annealing method and used as oxygen electrodes. The RuO_2_ nanoparticles (5–10 nm) are uniformly anchored in the three-dimensional B-HRG continuous electric network. The RuO_2_-B-HRG aerogel possesses a large specific surface area (287.211 m^2^ g^−1^) and numerous mesopores and micropores. The pores facilitate electrolyte impregnation and oxygen diffusion, and they provide greatly increased accommodation space for the discharge products. Electrochemical tests show that the RuO_2_-B-HRG/KB enables the electrode overpotential to decrease, and the rate capability and the cycling stability are enhanced compared with pure HRG. The enhanced performance is ascribed to the bifunctional catalytic activity of RuO_2_-B-HRG and its unique three-dimensional porous architecture. The method is proved to be an effective strategy to combine porous carbon materials and nanoscale catalysts as electrodes for Li–O_2_ batteries.

## Abstract 2

Hierarchically porous RuO_2_-B-HRG is a great bifunctional catalyst and effectively improve the performance of non-aqueous Li–O_2_ batteries.

## Graphical abstract

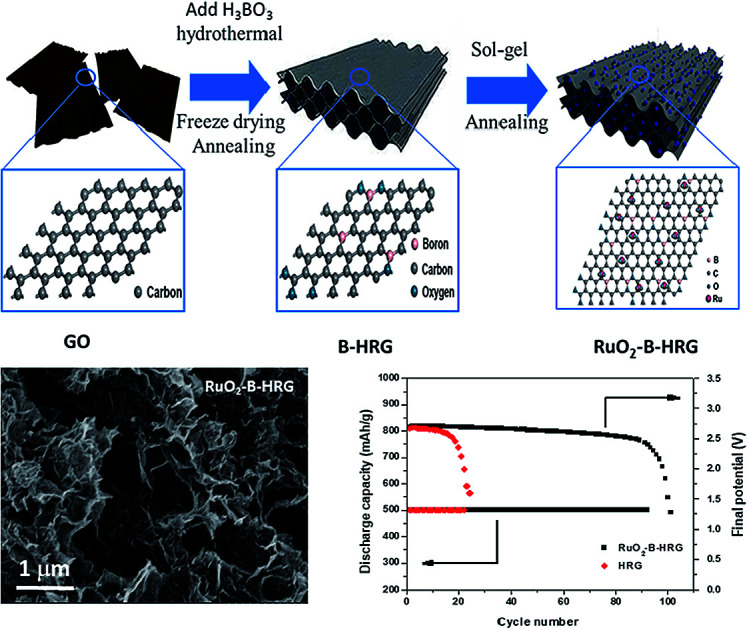

## 1. Introduction

The increasing requirements for sustainable energy demand the development of high-performance power sources with a high energy density and long cycle lives.^1^ Despite conventional Li-ion batteries having already been widely applied in the marketplace, these batteries are still limited by their insufficient energy density, usually below 200 W h kg^−1^.^2–4^ Li–O_2_ batteries have been considered a promising alternative due to their ultra-high theoretical energy density of 3505 W h kg^−1^, which is far more than that of Li-ion batteries.

Li–O_2_ batteries are first introduced in 1996, and are based on the reaction: 2Li + O_2_ ↔ Li_2_O_2_.^5^ More recently, remarkable progress in Li–O_2_ batteries has been made.^6,7^ Nevertheless, these batteries still face tough challenges before their practical application, such as degradation of the electrolyte, high discharge/charge overpotential and poor cycle stability.^8,9^ In Li–O_2_ batteries, the oxygen electrode is the main site for oxygen reduction and evolution reactions, which are vital to achieve high energy efficiency and cyclability.^10,11^ Generally, the cathode consists of porous conductive matrices (usually carbon materials), catalysts and a polymer binder. Among all kinds of carbon, graphene-related materials have been most widely used as cathode materials owning to their theoretically high electrical conductivity and good ORR catalytic activity. Moreover, graphene can be easily modified to enhance its properties or to endow it with new properties on demand. For instance, the chemical doping of graphene with various heteroatoms (N, B, S, and P)^12–15^ or the construction of a 3D porous structure aerogel^16,17^ both can provide enhanced performance. Recently, combining the advantage of boron-doping with a graphene aerogel structure, a simple one-pot hydrothermal method is adopted to prepare a 3D boron-doped graphene aerogel framework (B-HRG).^18–20^ Obviously, the B-HRG possesses a large surface area, a high electrical conductivity, and good ORR activity. Notably, loading catalysts, such as noble metals and mental oxide onto carbon materials has been an approach to enhance cathodic reaction kinetics for low overpotentials and high energy efficiency. To date, various electrocatalysts, including noble metals (Ru, Au, Pd, and Pt),^21–24^ transition metals and their oxides (RuO_2_, MnO_2_, Co_3_O_4_, and NiO),^25–29^ have been intensively explored to improve the performance of Li–O_2_ batteries. Particularly, RuO_2_ nanoparticles have been reported to exhibit excellent OER activities and drastically reduce the charge potentials of Li–O_2_ batteries.^30,31^

Herein, we fabricated ruthenium oxide modified hierarchically porous boron-doped graphene aerogels as cathode catalyst for Li–O_2_ batteries and used RuO_2_-B-HRG/KB as a cathode. This enabled a non-aqueous Li–O_2_ battery to obtain a long-term cycle and lower overpotential than it would have otherwise. When the capacity is limited at 500 mA h g^−1^, a stable cycling over 90 times as well as the lower overpotential of 0.5 V in the charge process can be achieved. This work will expound the design of bifunctional catalysts and enable high reversible capacity and the high-rate cycling stability of Li–O_2_ batteries.

## 2. Experimental

### 2.1 Synthesis of RuO_2_-B-HRG composite

Natural graphite powder (carbon content: 99.9%), hydrogen peroxide (H_2_O_2_, 30%), sulfuric acid (H_2_SO_4_, 98%), potassium permanganate (KMnO_4_), sodium nitrate (NaNO_3_), hydrochloric acid (HCl, 37%), boric acid (H_3_BO_3_), ruthenium(iii) chloride hydrate (RuCl_3_·*x*H_2_O), sodium hydroxide (NaOH) were obtained from Sinopharm Chemical Reagent Co., Ltd. All of these reagents were in the analytical purity grade and were used without further purification. Deionized water was used throughout the experiments.

**Fig. 1:**
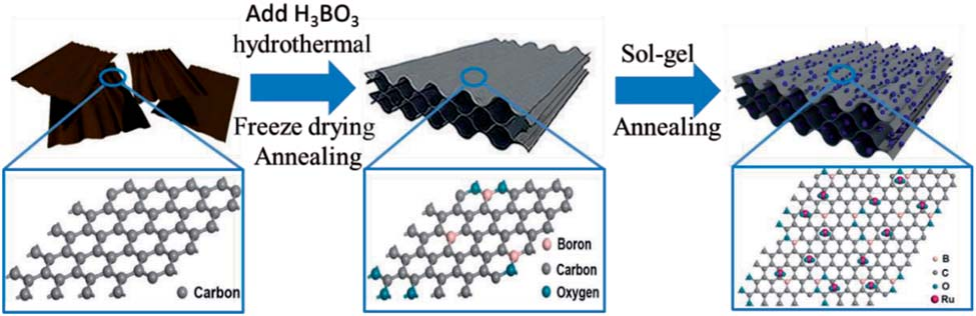
Schematic of the fabrication process of RuO_2_-B-HRG composite and diagram of the structure of atoms on a single layer for the GO, B-HRG, RuO_2_-B-HRG.

A synthesis scheme for ruthenium oxide modified boron-doped graphene aerogel composites is shown in Fig. 1. Graphite oxide (GO) was fabricated from natural graphite powder by a modified Hummers method.^32^ After being purified by dialysis, the newly prepared GO was treated by ultrasonication for an hour and then diluted to a 2 mg mL^−1^ colloidal suspension with deionized water. B-HRG was synthesized *via* a typical hydrothermal process. In detail, 50 mL graphite oxide (GO) was dispersed in 35 mL deionized (DI) water under sonication for 1 h. Then 500 mg boric acid was added into the GO solution, and the mixture was stirred for 1 h. Then the boric acid–GO solution was transformed in a 100 mL Teflon-lined stainless steel autoclave and hydrothermally treated at 180 °C for 12 h. After cooling naturally, the cylindrical B-HRG hydrogel was removed and immersed in deionized water for 24 h to remove the residue. The aerogel was procured through freeze-drying to maintain its porous structure. Then it was transferred into a quartz boat and placed in a tubular furnace and heated at 800 °C for 3 h under argon to removing the excess B_2_O_3_. Next, the B-HRG samples were soaked in a RuCl_3_·*x*H_2_O solution, to allow loading of Ru^3+^ on their surface, which was then converted to ruthenium hydroxide by adding 0.1 M NaOH to pH > 7. The product was collected by centrifugation washed several times with ethanol, and oven-dried at 80 °C. Finally, the products were annealed at 150 °C for 2 h under oxygen to obtain RuO_2_-B-HRG composite. For comparison, the HRG aerogel was prepared under the same experimental conditions but without addition of boric acid and RuCl_3_·*x*H_2_O.

### 2.2 Materials characterization

The crystal structure of the as-prepared sample was examined by X-ray diffraction (XRD, D8-Advance X-ray diffractometer, Cu Kα, *λ* = 1.5406 Å) in the 2*θ* range of 10–80°. The Brunauer–Emmett–Teller (BET) technique was used to characterize specific surface area and pore volumes by using a Micromeritics Tristar 3000. And the samples were degassed for 3 h at 300 °C under vacuum before surface area measurements. The morphology of the samples was characterized by field emission scanning electron microscopy (FE-SEM, Hitachi S-4800) and high resolution transmission electron microscopy (HR-TEM, JEM-2100F). Energy dispersive X-ray spectrometry (EDX) mapping were carried out to reveal the distribution of composition. A Kratos Axis Ultra X-ray photoelectron spectrometer with Al Kα source was utilized to study the chemical composition of the synthesized sample.

### 2.3 Electrochemical measurement

For Li–O_2_ battery, the cathodes were prepared by mixing as-synthesized composite, Ketjen black (KB), with polytetrafluoroethylene (PTFE) in the weight ratio of 6 : 3 : 1 onto a nickel foam current collector with a diameter of 12 mm. These samples were then dried at 80 °C for 12 h in a dry oven to remove the solvent. The total mass loading of the composite, KB and PVDF was approximately 0.6 mg cm^−2^. Then, the non-aqueous Li–O_2_ cells were assembled in an argon-filled glove box (H_2_O ≤ 1 ppm) using Swagelok batteries with an air window of 78.5 mm^2^, including an O_2_ electrode, a Celgard 3500 membrane and a 1 M lithium bis(trifluoromethanesolphonyl) imide (LiTFSI, Sigma-Aldrich, 99.95%) in tetraethylene glycol dimethoxyethane (TEGDME, Sigma-Aldrich, 99%) electrolyte, a Li foil anode.

The charge/discharge test of cells were conducted on a Land cycler (Wuhan Jinnuo Electronic Co. Ltd.) in the voltage range of 2.2–4.3 V (*vs.* Li^+^/Li) at different current densities. The specific capacity is based on the amount of whole composite and KB. The cyclic voltammograms (CVs) were conducted within 2.2–4.3 V at a sweep rate of 0.2 mV s^−1^ on CHI 660C electrochemistry workstation (Shanghai Chenhua, China). Electrochemical impedance spectroscopy (EIS) measurements were carried out on a CHI 660C electrochemistry workstation (Shanghai Chenhua, China) in a frequency range of 0.1–1 × 10^5^ Hz with an amplitude of 5 mV. All of the tests were carried out in an oxygen-filled glove box at room temperature. Fourier-transform infrared reflection (FTIR) measurement was carried out on a Thermo Fisher Nicolet 6700 FTIR spectrometer to study the cathode compositions after discharge and charge. To obtain the cathode after the test, the battery was disassembled in the argon glove box, and the cathode was rinsed with dimethyl carbonate (DMC) to remove the residual lithium salt over the cathode surface, and then dried at room temperature.

## 3. Results and discussions

### 3.1 Morphology and structure of the 3D-RuO_2_-B-HRG aerogels

**Fig. 2:**
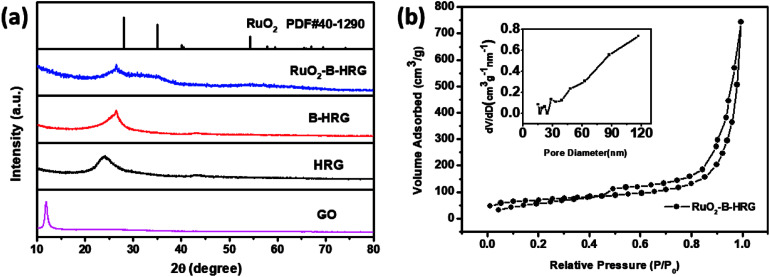
(a) XRD patterns of GO, HRG, B-HRG, RuO_2_-B-HRG. (b) Nitrogen adsorption–desorption isotherms and the pore size distribution curves (insert) of RuO_2_-B-HRG.

To confirm the formation of the desired phase in the materials, the XRD pattern for the GO, HRG, B-HRG and RuO_2_-B-HRG are shown in Fig. 2a. A strong and sharp diffraction peak at 11° belongs to the (002) planes of the GO. After facile hydrothermal treatment and pyrolysis, the sharp peak at 11° disappears while a new broad diffraction peak at 25° appears in the XRD pattern of HRG and B-HRG, confirming the successful conversion of GO to graphene. The shift from 24.2° to 26.5° in 2*θ* between HRG and B-HRG shows the boron doping into a hexagonal crystalline structure.^33^ After sol–gel method and subsequent low temperature annealing, three broad diffraction (2*θ* = 28°, 35°, 54°) appear, corresponding to (110), (101), and (211) of RuO_2_ respectively. These peaks were not observed in the pristine HRG. The peaks of as-prepared RuO_2_ were weak owing to the low annealing temperature.

Nitrogen adsorption/desorption measurements (Fig. 2b) were conducted to obtain the specific surface areas and pore volumes. The composites were found to have type-IV isotherms and an obvious H3 hysteresis loop with relative pressure in the range of 0.5–1.0, indicating the existence of a mesoporous structure. The specific surface areas and pore volume of RuO_2_-B-HRG were measured to be 287.211 m^2^ g^−1^ and 1.093 cm^3^ g^−1^, respectively. For the reference, the specific surface areas of the pristine-B-HRG and HRG samples were 502 and 589 m^2^ g^−1^, respectively (see Fig. S1a†). The lower surface area of RuO_2_-B-HRG can be attributed to the dense RuO_2_ coating. Meanwhile, it has broad pore size distribution including mesoporous and macroporous (inset in the Fig. 2b). The mesoporous structure is beneficial for oxygen transmission and electrode infiltration while the macroporous structure can provide enough buffer space for the discharged products.

**Fig. 3:**
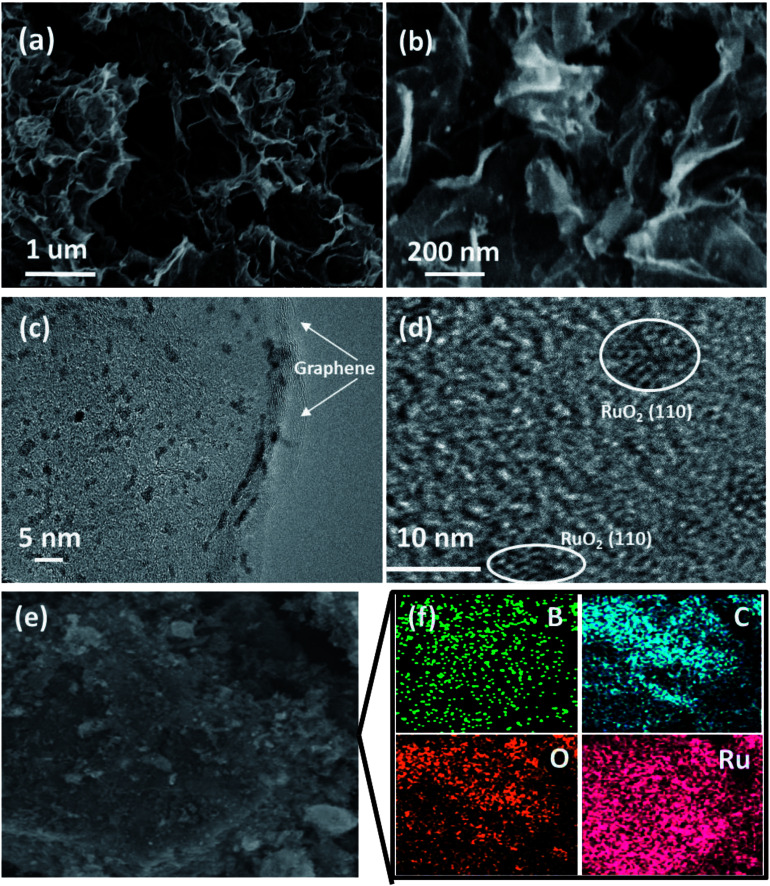
(a) Low-magnification SEM image of RuO_2_-B-HRG. (b) High-magnification SEM image of RuO_2_-B-HRG. (c) and (d) High-resolution TEM image of RuO_2_-B-HRG. (e) Low-magnification SEM image of RuO_2_-B-HRG. (f) Image of RuO_2_-B-HRG with the corresponding elemental mapping of (c) B, (b) C, (e) O, (f) Ru.

Scanning electron microscopy (SEM) images of the RuO_2_-B-HRG composites clearly revealed that countless pieces of graphene sheets stack to form a loose network structure with pore diameters from tens of nanometers to several micrometers (Fig. 3a), which agrees well with the B-HRG and HRG structure (Fig. S2†). Upon loading the aerogels with RuO_2_, some tiny nanoparticles were found on the surface and the inner pores of the aerogels (Fig. 2b). These results were in agreement with the Brunauer–Emmett–Teller (BET) results such as the pore size, pore distribution, and specific surface area of the samples. Transmission electron microscopy (TEM) images of as-obtained RuO_2_-B-HRG showed that the nanoparticles were uniformly dispersed on the B-HRG micro-sheets. High-resolution TEM images showed the obvious (002) plane of graphitic carbon (Fig. 3c) and the RuO_2_ nanoparticles with crystal lattices (110) (Fig. 3d). We used energy-dispersive X-ray (EDX) mapping to further investigate the distribution and composition of the composite. EDX maps of the elements B, C, O, and Ru and the composite are shown in Fig. 3f. The B, C, Ru and O elements were uniformly distributed on the whole samples. The relative element content (At%) of B, C, O and Ru in the hybrid materials were measured by Energy Dispersive Spectrometer (EDS), which yielded results of 1.95%, 58.08%, 19.22%, 19%, respectively.

**Fig. 4:**
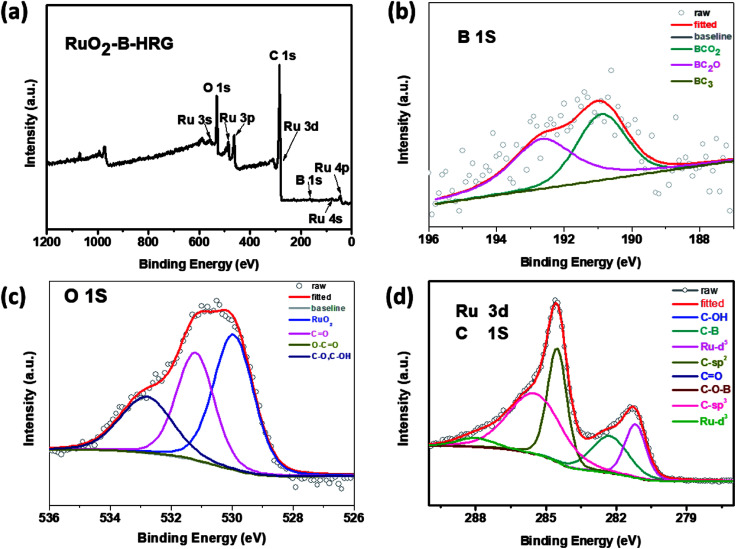
(a) XPS spectra for RuO_2_-B-HRG. (b) B 1s XPS curves. (c) O 1s XPS spectra. (d) C 1s and Ru 3d XPS curves of RuO_2_-B-HRG.

The chemical composition and binding state were investigated by X-ray photoelectron spectroscopy (XPS). Through the full range XPS analysis in Fig. 4a, the elements B, C, O and Ru were detected from the RuO_2_-B-HRG composite. XPS analysis Fig. 4b indicated that boron atoms were successfully incorporated into the HRG matrix *via* the high temperature thermal treatment. This was shown by the three fitted peaks at 192.3, 191.9 and 189.9 eV, which were ascribed to –BCO_2_, –BC_2_O, and –BC_3_ bonds, respectively. The high resolution O 1s spectrum (Fig. 4c) of composites was divided into four peaks located at 529.3, 531.5, 532 and 533.2 eV assigned to RuO_2_, O–C=O, C=O, and C–O/C–OH, respectively. The strong peak located at 529.3 eV suggests the existence of RuO_2_, not metal Ru. In the spectrum, the C 1s peak of RuO_2_-B-HRG (Fig. 4d) was fitted to four components. The strong peaks located at 284.8 and 285.6 eV were assigned to sp^2^ and sp^3^-bonded carbon atoms, respectively. The peak around 287 eV showed the existence of carbon–oxygen components, such as C–O, CO. A weak peak at 290.1 eV corresponds to the formation of the C–O–B of B-HRG. The Ru 3d^5/2^ peak at 281.1 eV (Fig. 4d) was clearly seen, corresponding to the binding energy of Ru^4+^, further suggesting the presence of RuO_2_ in the composite.

### 3.2 Electrochemical performance of the RuO_2_-B-HRG

**Fig. 5:**
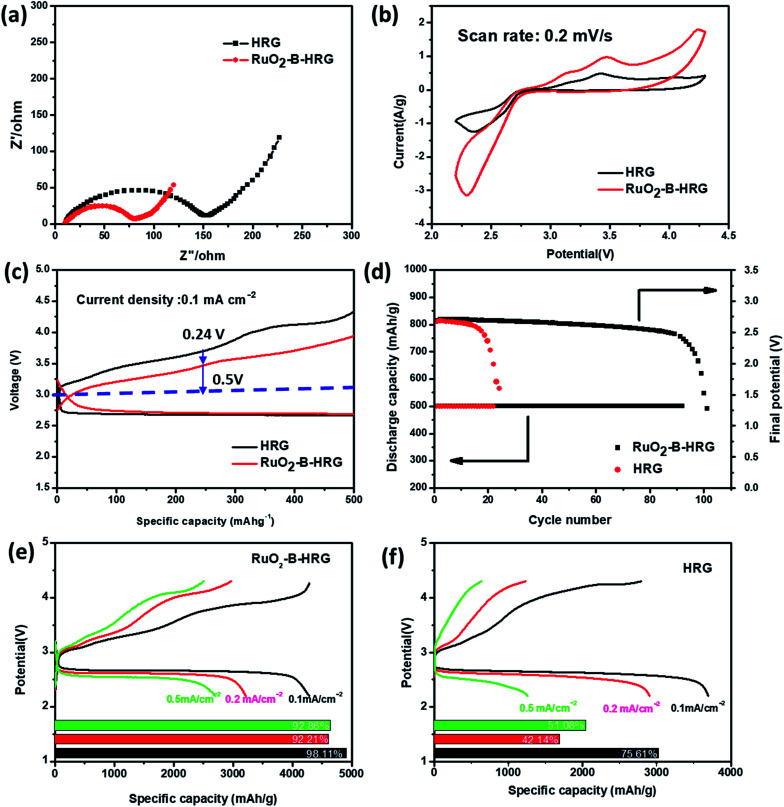
(a) Electrochemical impedance spectra of the RuO_2_-B-HRG/KB and HRG/KB electrodes in the first cycle. (b) Cyclic voltammetry curves of the RuO_2_-B-HRG/KB electrode at scan rate of 0.2 mV s^−1^. (c) Initial galvanostatic discharge–charge curves of RuO_2_-B-HRG/KB and HRG/KB at a fixed capacity of 500 mA hg^−1^. (d) Cycling stability of the RuO_2_-B-HRG/KB electrode based Li–O_2_ batteries under a specific capacity limit of 500 mA h g^−1^ at 0.1 mA cm^−2^ and the plots of cutoff voltage *vs.* cycle number. (e) Initial discharge–charge curves of RuO_2_-B-HRG/KB at different density and corresponding coulombic efficiencies (inset Fig. 5e). (f) Initial discharge–charge curves of HRG/KB at different density and corresponding coulombic efficiencies (inset Fig. 5f).

Electrochemical impedance spectroscopy (EIS) measurements are performed to further evaluate the electronic conductivity. It is known that, the diameter of a semi-circle in the high frequency region of the EIS spectra corresponds to charge transfer resistance. The results in Fig. 5a demonstrated that the RuO_2_-B-HRG cathode exhibits an obvious smaller charge transfer resistance than the HRG, suggesting the RuO_2_-B-HRG cathode can process higher electrical conductivity than other cathodes, thus assisting in reducing the polarization.

In order to evaluate the electrocatalytic activity (ORR and OER) and cycle stability of the as-prepared RuO_2_-B-HRG composition, batteries based on the RuO_2_-B-HRG samples were examined and pristine HRG was employed for comparison. The electrocatalytic activity of RuO_2_-B-HRG was detected using cyclic voltammetry (CV) at 0.2 mV s^−1^ (Fig. 5b). Compared with a bare HRG cathode, the RuO_2_-B-HRG cathode exhibited a higher peak current density in both cathodic and anodic processes in the first cycle, demonstrating its enhanced kinetics in ORR and OER. Specifically, the ORR onset potential of RuO_2_-B-HRG compositions was positive than that of HRG. Meanwhile, the cathode also presented a more negative onset OER potential. The obvious peak at 3.4 V could be seen both in the HRG cathode and in the RuO_2_-B-HRG cathode, corresponding to the oxidation of LiO_2_. The enhanced ORR/OER kinetics tended to present outstanding performance, such as higher reversible capacity, lower overpotential and better rate capacity.

Li–O_2_ battery performance based on RuO_2_-B-HRG was measured using galvanostatic charge–discharge tests at 500 mA g^−1^ within a potential window from 2.2 to 4.3 V, as shown in Fig. 5c. In the charge process, the HRG cathode had a large overpotential of 0.76 V while the RuO_2_-B-HRG cathode only had 0.5 V, in line with the EIS and CV measurements. Furthermore, RuO_2_-B-HRG also had lower overpotential than HRG in the discharge process, which was attributed to the bifunctional catalytic activity of the composition.

The cycling stability of the Li–O_2_ batteries was investigated and the results are shown in Fig. 5d. The cycling measurements were performed at 0.1 mA cm^−2^ with a limited discharge capacity of 500 mA h g^−1^. The specific discharge–charge curves of the electrode at different cycle counts are given in Fig S3.† As shown in Fig. 5d, the cell with the HRG electrode operated stably for about 20 cycles until the terminal voltage decreased below 2 V while the RuO_2_-B-HRG electrode worked stably for more than 90 cycles between 2 V and 4.3 V, demonstrating almost quadruple the stability of the former. The improved cycling stability is mainly attributed to the synergistic effect of excellent electrical conductivity, ORR/OER activity and three-dimensional porous architecture. The RuO_2_-B-HRG cathode can deliver an initial discharge capacity of 4300 mA h g^−1^ at a current density of 0.1 mA cm^− 2^, which is larger than pure HRG materials (Fig. 5e and f). The electrode also shows good rate performance and excellent reversibility (Fig. 5e). Additionally, the coulombic efficiencies of RuO_2_-B-HRG electrode at different current densities are over 90%. Unfortunately, the coulombic efficiencies of the HRG/KB are around 50% with a large current density (0.2, 0.5 mA cm^−2^) (Fig. 5f).

**Fig. 6:**
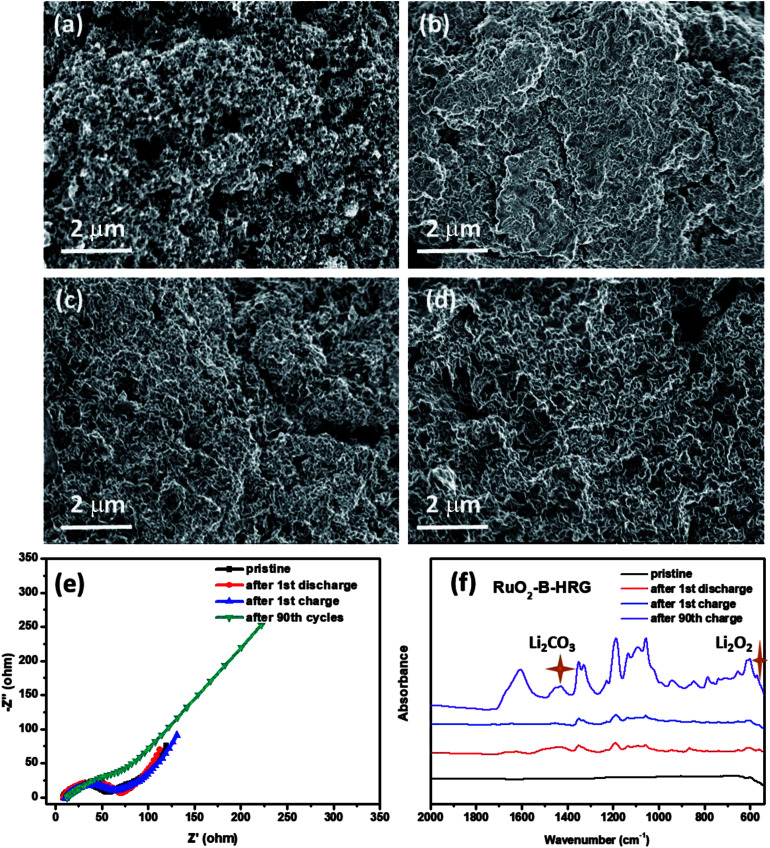
SEM images of the RuO_2_-B-HRG/KB electrode (a) pristine, (b) after the first discharge process, (c) after the first charge process. (d) After 90 cycles. (e) Electrochemical impedance spectra and (f) FTIR spectra of the RuO_2_-B-HRG/KB electrode in the first cycle at current density of 0.05 mA cm^−2^.

To further explore the reversibility of the battery with a RuO_2_-B-HRG electrode, SEM, EIS and FTIR were used to investigate the discharge products and surface status of the electrodes which went through the first cycle given in Fig. 6. The SEM, EIS and FTIR spectrograms of the HRG/KB electrode were showed in Fig S3 and S4.† The pristine electrode made up of RuO_2_-B-HRG with a porous structure and KB nanoparticles (Fig. 6a), showed a myriad of holes with various sizes. After the first full discharge, the holes on the electrode surface were completely covered by an uneven film (Fig. 6b), which might block the transmission of electrons and oxygen. However, after the first charge, the product film disappeared and the porous structure on the electrode surface recovered, just like the pristine status (Fig. 6c), implying the decomposition of the product and the good reversibility of the RuO_2_-B-HRG electrode. However, the surface status of the electrode after the 90^th^ charge is showed in Fig. 6d, where the product film again exists and the porous structure is hard to see. It could be one of the reasons for the termination of cycle. To further investigate the changes of discharged products in the discharge/charge process, EIS spectrograms were obtained (Fig. 6e). After the first cycle of discharging, the composition electrode exhibited a bigger charge transfer resistance than did the pristine sample, which shows generation of non-conducting discharged products. After the first charge, the resistance is basically the same as for the pristine, showing the complete decomposition of the discharged products. The resistance of RuO_2_-B-HRG is obviously larger than that of the pristine sample due to the incomplete decomposition of discharge products, which lead to the increased overpotential in the cycle process. In order to identify the composition of the discharge products, the FTIR spectra is showed in Fig. 6f. It could be observed that the characteristic IR peaks for Li_2_O_2_ appeared after the first discharge and nearly vanished again in the curve after the charge process. But beyond that, there were some peaks assigned to Li_2_CO_3_ which appeared after discharge and then disappeared after charge. The Li_2_CO_3_ probably mainly came from the slight decomposition of the electrolyte and the inevitable reaction between Li_2_O_2_ and CO_2_ in the air during the FTIR test. The IR of 90 cycles showed some sharp peaks belonging to Li_2_O_2_ and Li_2_CO_3_. These results led us to conclude that Li_2_O_2_ is the dominant discharge product after the discharge process for the RuO_2_-B-HRG/KB electrode. Meanwhile, the byproducts such as Li_2_CO_3_ increase along with the increasing cycle.

## 4. Conclusions

In summary, ruthenium oxide modified hierarchically porous boron-doped graphene aerogels (RuO_2_-B-HRG) can be successfully synthesized by sol–gel method and low temperature annealing. The as-prepared three-dimensional porous B-HRG possessed a high electrical conductivity and improved activities for the ORR, meanwhile, the large surface area and porous structure of B-HRG not only enabled a low amount of noble metal catalyst into the cathode in a well-dispersed distribution but also provided enough space to store a large amount of Li_2_O_2_ formed during discharge. The exposed RuO_2_ nanoparticles can catalyze electrochemical reactions, especially oxygen evolution reactions. The RuO_2_-B-HRG hybrids explored as bifunctional electrocatalysts for the air electrode in Li–O_2_ batteries show excellent electrochemical performance and rate capability. These batteries can be fully discharged/charged over 90 cycles at a fixed capacity of 500 mA h g^−1^. They can remarkably reduce charge potentials to 3.7 V at a current density of 0.1 mA cm^−2^ and have a capacity of 4300 mA h g^−1^, higher than that of HRG. Furthermore, RuO_2_-B-HRG can guide the reversible growth or decomposition of Li_2_O_2_ into a thin-film form. The superior performance of these batteries is attributed to the combination of outstanding catalyst and reasonable structure. This investigation suggests that the hierarchically porous RuO_2_-B-HRG are a promising stable cathode, and provides hints on the design and construction of nano-composite catalysts for cathodes of Li–O_2_ batteries.

## Conflicts of interest

There are no conflicts to declare.

## Supplementary Material

RA-008-C8RA08763F-s001
