# Supplementary material for: Ruthenium oxide modified hierarchically porous boron-doped graphene aerogels as oxygen electrodes for lithium–oxygen batteries
Source: RSC Adv. 2018 Nov 29;8(70):39829–36. doi: 10.1039/c8ra08763f (PMC9091283; doi:10.1039/c8ra08763f)
Supplement: RA-008-C8RA08763F-s001 [file RA-008-C8RA08763F-s001.pdf]

Electronic Supplementary Material (ESI) for RSC Advances.
This journal is © The Royal Society of Chemistry 2018

# Ruthenium oxide modified hierarchically porous boron-doped graphene aerogels as oxygen electrode for lithium oxygen batteries

Xiuhui Zhang[a], Chunguang Chen[a], Xiang Chen[b], Tie Liu[a], Mengmeng Liu[b], Congcong Zhang[b], Tao, Huang[b], Aishui Yu[a,b,*]

[a]Department of Chemistry, Shanghai Key Laboratory of Molecular Catalysis and Innovative Materials, Institute of New Energy, Collaborative Innovation Center of Chemistry for Energy Materials, Fudan University, Shanghai 200433, China.

[b]Laboratory of Advanced Materials, Shanghai Key Laboratory of Molecular Catalysis and Innovative Materials, Institute of New Energy, Collaborative Innovation Center of Chemistry for Energy Materials, Fudan University, Shanghai 200433, China.

[*]E-mail: asyu@fudan.edu.cn

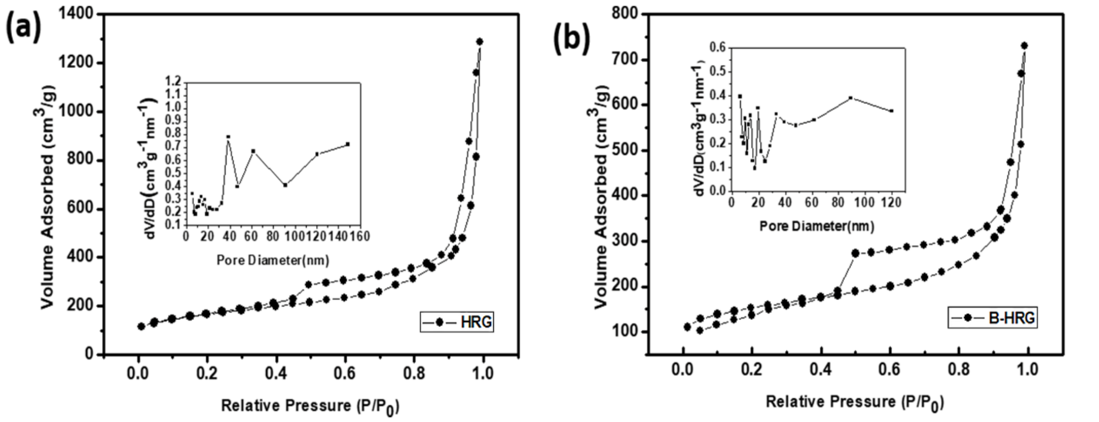


**Fig. S1.** Nitrogen adsorption-desorption isotherms and the pore size distribution curves (insert) of HRG (a) and B-HRG (b).

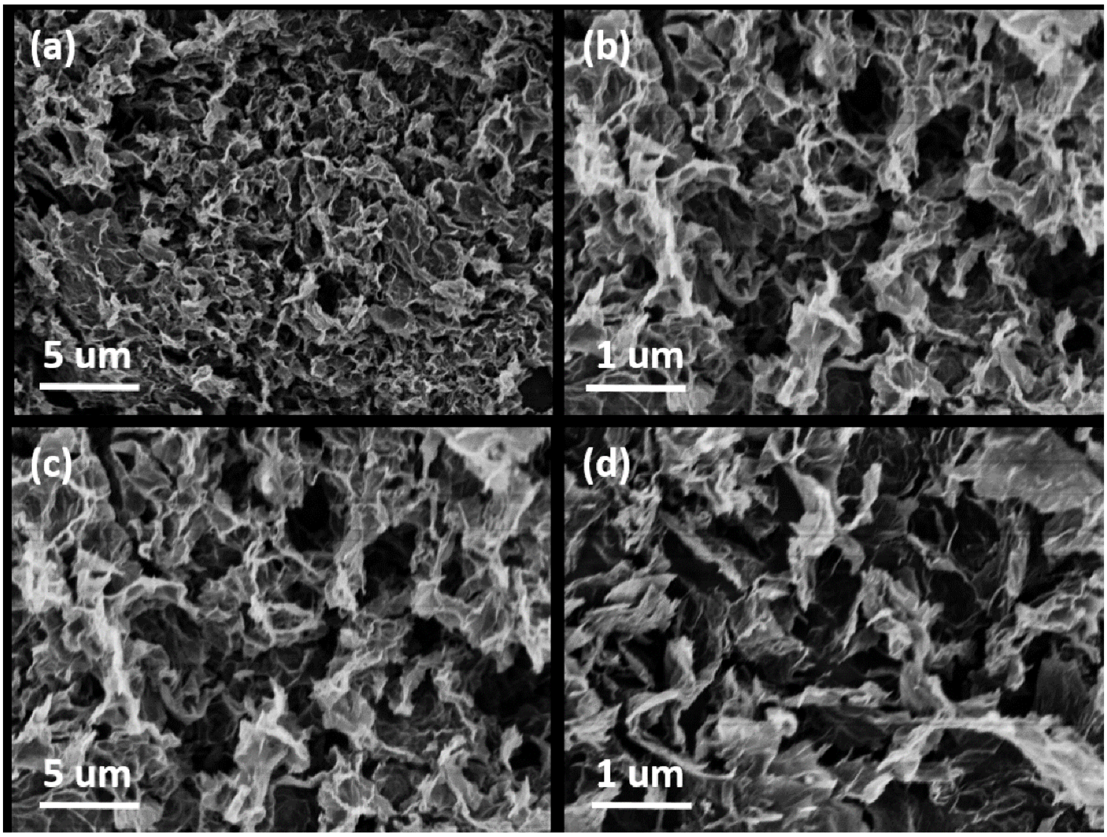


**Fig. S2.** Low-magnification SEM images of (a) (b) HRG, and (d) (e) B-HRG.

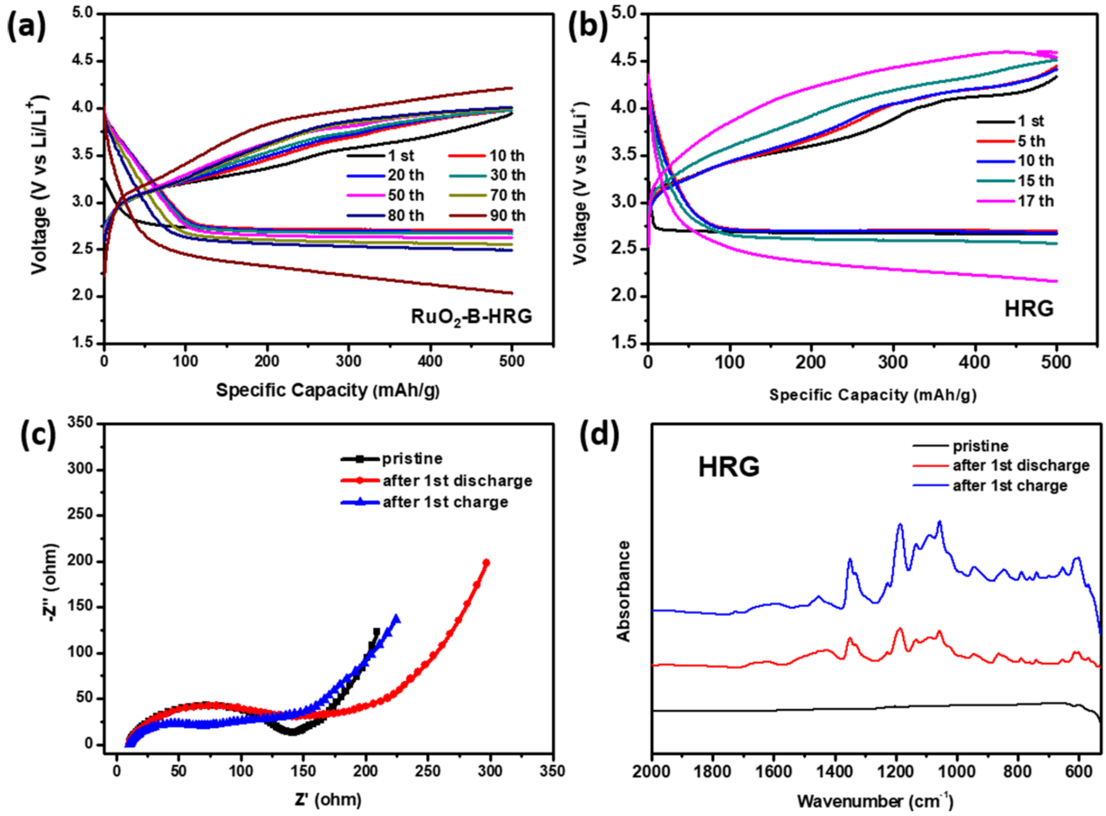


**Fig. S3.** (a) Rate capacities of the Li–$O_2$ battery based on HRG/KB at different current

densities. (b) the discharge-charge curves of HRG/KB electrode with a fixed capacity of 500 mAh $g^{-1}$ at a current density of 0.1 mA $cm^{-2}$.(c) Electrochemical impedance spectra and (d) FTIR spectra of HRG /KB electrode in the first cycle at current density of 0.05 mA/$cm^2$.

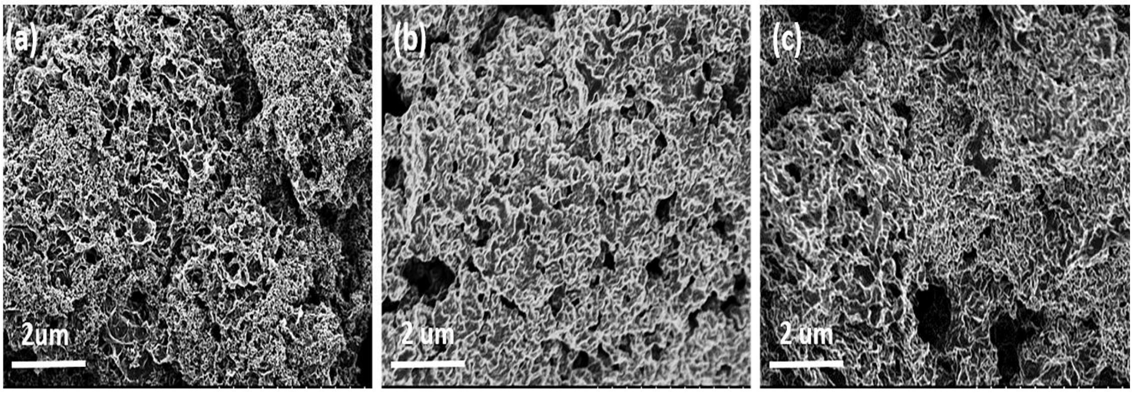


**Fig. S4.** SEM images of the HRG/KB electrode (a) pristine, (b) after the first discharge process, (c) after the first charge process.
